# Proposal for Tier-Based Resumption of Dental Practice Determined by COVID-19 Rate, Testing and COVID-19 Vaccination: A Narrative Perspective

**DOI:** 10.3390/jcm10102116

**Published:** 2021-05-14

**Authors:** Nima Farshidfar, Dana Jafarpour, Shahram Hamedani, Arkadiusz Dziedzic, Marta Tanasiewicz

**Affiliations:** 1 Student Research Committee, Shiraz University of Medical Sciences, Shiraz 71348-14336, Iran; 2 Faculty of Dentistry, McGill University, Montreal, QC H3A 1G1, Canada; dana.jafarpour@mail.mcgill.ca; 3 Oral and Dental Disease Research Center, School of Dentistry, Shiraz University of Medical Sciences, Shiraz 71956-15878, Iran; shahramhamedani@yahoo.com; 4 Department of Restorative Dentistry with Endodontics, Medical University of Silesia, 40-055 Katowice, Poland; martatanasiewicz@sum.edu.pl

**Keywords:** COVID-19, SARS-CoV-2, coronavirus, dental care, resumption, mitigating measures, vaccination, rapid testing

## Abstract

Since the emergence of the new coronavirus disease (COVID-19), profound alterations in general and specialist dental practice have been imposed to provide safe dental care. The guidelines introduced in response to the COVID-19 pandemic to mitigate healthcare disruption are inconsistent regarding the dental practice re-installation, particularly during a transitional time. Despite the successful mass vaccination campaigns rolled out in 2021, the presence of more than 80 genotypes of COVID-19, rapid neutralisation of antibodies within a short period of seropositivity, and the likelihood of recurrent infection raise some doubts on whether vaccination alone will provide long-term immunity against COVID-19 and its variants. Here, from this perspective, we aim to provide an initial proposal for dental services reinstallation, easily applicable in various care settings. We discuss the potential options for the transition of dental services, as well as challenges and opportunities to adapt to new circumstances after mass COVID-19 vaccination. The proposal of the universal three-tier system of dental services resumption, determined by regional COVID-19 rates, testing accessibility, and vaccination rollout has been presented. Following herd COVID-19 immunity enhancement, it would be prudent to confer various preventative measures until virus spread naturally diminishes or becomes less virulent. Based on modelling data, dental practices may not return to normal, routine operation even after global vaccination as there would still be a significant risk of outbreaks of infection. Variable, multi-level measures will still be required, depending on the local COVID-19 cases rate, to secure safe dental care provision, despite predicted success of vaccination agendas. This approach can be implemented by achievable, practical means as a part of risk assessment, altered work pattern, and re-arrange of dental surgery facilities. The adequate standard operating procedure, with the support of rapid point-of-care testing at workplace, would vastly intensify the uninterrupted recovery of the dental care sector.

## 1. Introduction

**Figure 1:**
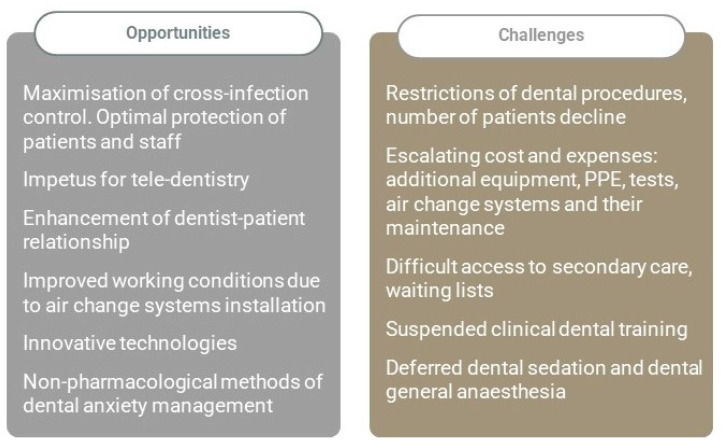
The impact of COVID-19 on the dental care sector. Potential opportunities and negative consequences.

The new coronavirus disease (COVID-19) pandemic has dramatically changed the main aspects of healthcare systems globally, including dental care provision on all levels of primary and secondary care [1,2]. This unprecedented phenomenon has triggered inevitable major workplace changes, global vaccination, as well as rapid testing innovations [3,4]. The consequences of severe acute respiratory syndrome coronavirus 2 (SARS-CoV-2) threat have given an impetus for innovative trends and technologies, as often happens when humanity faces a global challenge [5]. Besides its obvious negative impact, the COVID-19 pandemic has also created opportunities for the dental care sector (Figure 1). It is predicted that dental practice will survive the pandemic as altered as the rest of the healthcare sectors. Disruptive pandemic events can give sudden, overall positive impetus for acceleration of organizational changes.

Globally, the authorities have released safety protocols and guidelines to decrease the risk of SARS-CoV-2 transmission and help dental care providers control the spread of the virus during the first wave of the pandemic and the health services re-installation phase [4,6]. Accordingly, these heterogeneous recommendations constantly evolve, supporting medical and dental workforce by ensuring safe working conditions and subsequently, putting in place locally implemented measures, adequate to any dental settings. In particular, dental care workers (DCWs) who perform their duties in the proximity of upper respiratory tract and are usually exposed to air-borne infectious bio-aerosols, have to rely on specific protective measures and guidelines to minimise the risk of being infected [6]. They face a high risk of contracting COVID-19 because of exposure to potentially contaminated saliva and microdroplets during aerosol-generating dental procedures (AGPs) [2].

This perspective aims to broaden the outlook of dental practice management following immense COVID-19 vaccination campaigns and puts forward constructive suggestions for routine dental service resumption after vaccination has been implemented on a global scale. To date, relatively few proposals of structured reinstallation of dental care provision have been presented during the transitional period, in the light of subsequent pandemic waves. This is surprising, considering mass inoculation and recently developed modern modalities to contain COVID-19 in the form of rapid testing. Undoubtedly, the response to immunization and prevention of virus transmission is not only largely driven by vaccine distribution, but also by the emergence of new SARS-CoV-2 variants. To the best of our knowledge, this is the first report of the universal tiers-based proposal for dental services reinstallation after COVID-19 emergence.

## 2. Primary Measure: The Adequate Standard Operating Procedures (SOP) Comprising Vaccination Status

Health authorities such as the World Health Organization (WHO) [7], Centers for Disease Control and Prevention (CDC) [8], the American Dental Association (ADA) [9] and the United Kingdom National Health Service (UK NHS) [10] have introduced essential guidelines for dental practice during the COVID-19 outbreak. These rigorous region-specific or national mitigating measures have been proposed to protect the staff and patients simultaneously, and to decrease the spread of COVID-19 by offering a consistent and scientifically proven series of steps. However, the concern remains whether professionals will disregard proposed guidelines after widespread vaccination programs have been completed, and also if the dental practice could then return to the way it had been operating before the pandemic.

Considering the prospect of COVID-19 vaccination, there is ongoing dispute regarding how dental practice would change if population-wide immunity against COVID-19 is achieved. Currently, limited recommendations from regulatory bodies exist regarding the safe resumption of routine dental care services during recovery period in 2021 [7,8,9,10]. It is predicted that the shift from strict Standard Operating Procedure (SOP) for dental management and the use of enhanced personal protective equipment (PPE) towards a more routine approach will occur, based on vaccination success and available rapid testing. The specifically adjusted SOP in dentistry aims to provide uninterrupted and efficient safe working environment while reducing the risk of COVID-19 infection. It highlights various crucial aspects to secure health and safety in the workplace.

**Figure 2:**
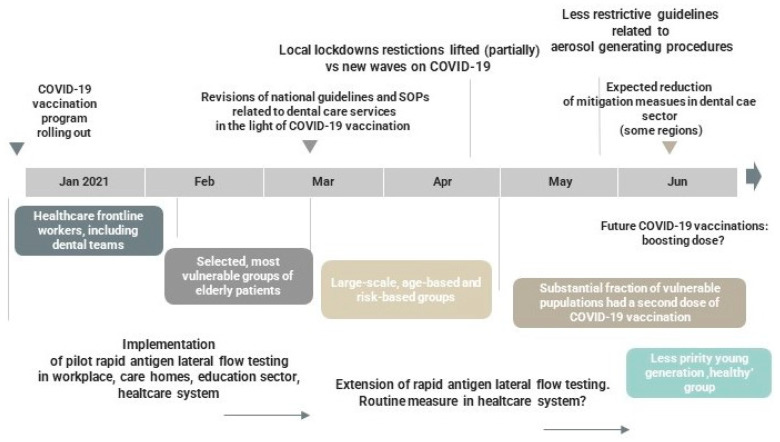
The predicted timescale of staged de-escalation and reinstallation of dental care services in 2021. Sequelae determined by COVID-19 mass vaccination.

The timeframe for the reinstallation of dental care since the pandemic outbreak is yet unpredictable, depending on potential new COVID-19 waves, virus variants, access to extended vaccination, cohorts’ immune response, and ‘self-adjustment’ of the dental sector to the new clinical reality. The fluctuating COVID-19 rates are predicted to escalate the conundrum of dentistry re-establishment across the world. Therefore, rolling out rapid in vitro diagnostics for DCWs at the workplace on a larger scale appears to be a viable option to detect asymptomatic cases and subsequently amplify the service recovery. It might be, however, challenging and compromised by logistic and/or financial constraints (Figure 2). It is predicted that dental services based in the university hospitals, community clinics or dental schools, and multi-unit dental corporate bodies would face a quicker adaptation and ‘re-invention’ of the new reality, compared to small general dental practices.

Even though evidence from studies regarding mass vaccination campaigns is promising [11], the presence of more than 80 genotypes of the COVID-19 coronavirus, neutralising antibodies with a short period of seropositivity, and the likelihood of re-infection have raised doubts whether vaccination will lead to long-term immunity [12]. A recent study demonstrated that the newly emerged SARS-CoV-2 variants B.1.1.7 (UK), B.1.351 (South Africa), and P.1 (Brazil) can escape immune response induced by vaccination or infection, with serious implications for pandemic mitigation [13]. Predominantly, it has been shown that the serological levels of immunoglobulin do not accurately correlate with the shedding of virus particles and the risk of transmissibility [12]. The homogeneity of infected populations in a particular timeframe might not be achieved due to the short-term immunity against the virus. Consequently, there might be no long-term, continuous herd immunity due to the possibility of re-infection, which might take place even with the existence of neutralising immunoglobulins [14]. Individuals need to be reminded to attend for the second vaccine dose, as missing this could lead to spreading, mutating of the virus, and potentially to becoming vaccine-resistant [15].

A concern regarding the firstly introduced COVID-19 vaccines is the necessity of administering two doses. Moreover, as new vaccines begin to emerge, this challenge persists and may particularly hinder the sufficient supply to less developed countries [12,14]. The obvious inequalities associated with the access to COVID-19 vaccine, and its disproportionate distribution across the world (for various reasons) may considerably compromise combating COVID-19. These factors are likely to increase the equity gap between countries and delay the eradication pandemic. Hence, the continuation of basic preventive measures for DCWs should be maintained until further evidence related to the virulence rate and its pathogenicity is revealed [14]. Prevention is particularly important in dentistry, where mutual contact between the DCWs and patients is unavoidable.

## 3. Optimizing Risk-Reduction: Teleconsultations and Chair-Side Tests Following Immunity Enhancement

Since 2020, remote teleconsultations have become a standard part of the SOP protocol to assess, diagnose, and protect DCWs, as well as to detect COVID-19 cases. It underpins the efforts to restore the normal pattern of clinical work. In fact, the COVID-19 pandemic may lead to a permanent re-organisation in dentistry with further development of new software for multi-purpose teledentistry [16,17]. Reduction of the risk of infection and protection of staff have been the main reasons for developing teledentistry. Virtual appointments, online consultations, and follow-ups, as well as the application of artificial intelligence to support clinical diagnosis have now been rendered practical through tele-dentistry [18]. In terms of risk mitigation and given the reduced risk of viral transmission using teledentistry, it is expected to become a pivotal, routine aspect of patient’ management following rapid testing to find out whether patients are infected or not [17]. A recent survey study has shown significant patient satisfaction towards using virtual consultations via teledentistry [19].

Hence, dental providers are encouraged to adopt telehealth as an optimal mode of consultation, initial examination and triaging during recovery time. It should be widely incorporated as a COVID-19 outbreak response, including specific SOP [20] and subsequently as a pillar of restored services. Moreover, consultations with the orthodontist during the orthodontic treatments and follow-up procedures can be easily performed via teledentistry. Recent studies have proposed a new concept of dental monitoring which integrates teledentistry with artificial intelligence in order to monitor orthodontic treatments in a semi-automatic manner [21]. Due to these substantial advantages of tele-dentistry in data collection, risk-assessment, saving of time, and providing better access to oral healthcare, this modern modality can eventually become an essential adjunct to the traditional approaches in dental practice [20]. Due to the ‘nature’ of dental profession, obviously they cannot substitute restorative or surgical services in people with active dental diseases and substantial needs of complex dental care.

**Figure 3:**
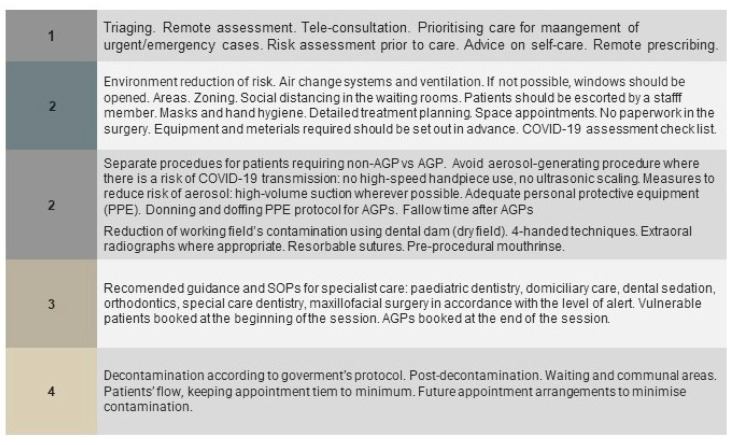
The essential elements of standard operating procedures applicable for the dental service.

Until now, although results have been promising as regards effective vaccination, it has been suggested that individuals should continue to aim for prevention and diagnosis of COVID-19 until a worldwide introduction of effective vaccine programs is in place (Figure 3). Given these circumstances, and taking into account the risk of asymptomatic carriers, it is rational to assume that ‘conventional’ dental practice might continue to expose DCWs to the risk of infection [22]. Therefore, fit-for-purpose SOP constitutes the core aspect of dental practice management to secure safe work environment for staff and patients.

### Rapid Antigen Tests: A Gamechanger?

Although teleconsultation and triaging may support the detection of symptomatic cases in the medical and dental sectors, reverse transcription-quantitative PCR (RT-qPCR) tests are the gold standard to diagnose COVID-19 [23,24]. Yet, the application of these laboratory tests is not feasible in dental clinics due to long waiting times for test results and lack of RT-qPCR testing facilities. Many rapid antigen qualitative tests such as commercial lateral flow assays have been developed for initial triage and rapid qualitative assessment of SARS-CoV-2 antigens [25]. These rapid lateral flow antigen tests (LFATs) can provide valid results within 10 to 20 min with sensitivity and specificity similar to quantitative immunoassays [26]. The opportunity to perform LFATs as rapid point-of-care tests (POCTs) outside the clinical laboratory is of great assistance, particularly in the clinical contexts where triaging patients is required. In general, LFATs mainly based on colloidal gold immunochromatography assay are intended for the qualitative detection of nucleocapsid antigens from human nasal and throat swabs, as well as sputum samples [26,27].

Considering the core priorities to combat a pandemic, it has been proposed that performing reliable and validated POCTs with acceptable sensitivity and specificity could potentially become a crucial element for triaging/screening all patients attending dental appointments. Particularly, chair-side LFATs can become a game changer on global scale. Rapid testing seems especially justified in the multi-unit clinical settings, i.e., hospital-based community medical/dental services, university hospitals and clinics. Besides standard nasal/pharyngeal swabs—because saliva is a relevant specimen for COVID-19 diagnosis and its collection is inexpensive, non-invasive, fast, and safe—accurate saliva-based POCT can help dental teams take the leading position in COVID-19 diagnosis, even in the post-vaccination era [24,28,29]. Employing more advanced diagnostics such as smartphone-based microfluidic systems could pave the way for straightforward pre-treatment diagnosis of other viruses in the dental setting [24].

Apart from key and obvious benefits, such as its simple use in clinical settings and its rapid results, LFATs have some disadvantages, including lower sensitivity compared to RT-qPCR and—as a result—potentially more false-negative results if SARS-CoV-2 antigen load in a sample is low [25,26,27]. However, the advantages of applying LFATs as an additional measure to protect DCWs while resuming the routine dental care prevail over their limitations (Table 1).

What is more, the positive ‘psychological effect’ of rapid COVID-19 testing in dental settings cannot be underestimated, enabling reassurance, and strengthening patients’/staff confidence [30]. As a result, this approach, although costly and logistically challenging in resource-limited areas, would improve the patient attendance rate and reduce the employee’s absence rate [30]. Most importantly, regular rapid LFATs are recommended as a routine protocol for staff members who provide special care dentistry for medically compromised individuals, persons with special needs (intellectual or developmental disabilities), and frail care home residents, to limit the treatment-related risk of SARS-CoV-2 infection [31].

Rapid testing in dentistry also depends on sufficient expertise, perception and attitude of managers/dental practice owners’, which account for their decisions. Primarily, LFATs constitute a particularly viable option to increase service capacity and decrease access inequalities in special care dentistry, as its provision was significantly reduced due to the pandemic [32]. Considering supply limits, rapid LFATs allow for more personalized care, prioritizing groups of patients with unmet healthcare needs. As persons with special needs present with generally poorer oral health, they are likely to require urgent dental care, and also are susceptible to COVID-19 contraction due to the high prevalence of infections in care institutions. This propagation of LFATs in dental and/or any other primary care settings, would considerably support public health ‘surveillance’ policy to monitor the trends of SARS-CoV-2 rate in asymptomatic local communities.

In the United Kingdom, LFATs have been already incorporated in primary care and care homes for staff and residents since the end of 2020 [31,33]. Moreover, LFATs are recommended for other occupational groups with increased risk of mass-spreading infection, e.g., in education sector. Recently, Conwey et al. [34] demonstrated feasibility of implementation a rapid screening and testing protocol in asymptomatic patients presented in dental surgery as response to the pandemic.

## 4. Will the COVID-19 Vaccines Modify PPE Use for High-Risk Procedures?

After the commencement of the mass vaccination campaigns in December 2020, an important issue could be the continued use of conventional vs. enhanced PPE in dental practice. Scientists predict that SARS-CoV-2 virus, which causes COVID-19, may outsmart immunity stimulation and it will eventually become an endemic strain, despite successful global vaccination in the future [35]. A recent modelling study [36] concluded that the vaccination for COVID-19 alone is unlikely to prevent further spread of the infection, to enable achievement of herd immunity, and to fully control the virus. The above-mentioned study predicts that the indicative R number will be still high, around 1.58 in the United Kingdom, even if the vaccine prevents a vast proportion of new infections [36]. Thus, even with mass-scale immunisation, the protective measures for routine clinical practice that were used pre-pandemic might not be sufficient, particularly during dental treatment involving aerosol-generating procedures (AGPs) creating potentially infectious aerosol [37]. This requires further scientific evidence regarding the virus pathogenicity, transferability, and rate of spreading.

To protect DCWs, it is imperative to choose suitable PPE according to the level of exposure to risk. Predominantly, the main concerns are costs, impact on the environment (waste), and physical discomfort, which reduce the necessary versatility, comfort, and vision needed for precise dental work [38]. Despite the intrinsic drawbacks of using traditional PPE (heat stress, lack of mobility, fall hazard, physiological effects, and anxiety), PPE has recently benefitted from high-tech developments [38], protecting DCWs adequately without compromising their comfort and capacity to perform clinical tasks.

Due to the anticipated reduction of AGPs in general, it is predicted that some dental specialities will introduce ‘AGPs-limited’ protocols. This may involve more common use of Hall (stainless steel crowns) and atraumatic restorative technique in paediatric dentistry, the usage of 3D intraoral technology instead of conventional impressions in prosthodontics and orthodontics, pre-procedural mouth rinse, disinfection of dentures and removable orthodontic appliances during their adjustment [39,40]. What is more, broader utilization of conscious dental sedation, instead of treatment under general anaesthesia in patients with special needs [40], as well as digital procedures in orthodontics [41] would become common standards in clinical practice.

Recent studies demonstrated that the materials used in PPE, especially soft surfaces, might act as carriers for the acquisition and transfer of pathogens after having been contaminated with them [38]. Additionally, the survival of infectious pathogens on PPE can expose DCWs and patients, particularly when PPE get damp or when protein-enriched biological waste is present on them [38]. Kasloff et al. [42] found significant variations in virus stability after drying SARS-CoV-2 over 21 days on the surfaces of commonly used PPE items and surfaces found in healthcare facilities. The viability of SARS-CoV-2 on non-porous and porous surfaces was between 4 (gloves) and 21 days (plastic, N-95, and N-100 masks) [42]. As SARS-CoV-2 has been found to persist on the surfaces of various types of PPE for several days [42], the use of superior textile materials, particularly in dental health care settings, should be considered an alternative to overcome the previously stated drawbacks of conventional PPE. These materials should also have both fluid-resistant and antimicrobial properties to reduce the probability of contamination, growth, and transmission of contagious pathogens on their surfaces [38]. Therefore, the reduction of additional, enhanced PPE use following inoculation and POC testing during and after the reinstallation phase appears paramount. This approach which is based on safety checks, will also have a positive impact on reduction of global carbon footprint.

Without widely applied and easy-to-be used rapid testing, the acquired herd immunity against COVID-19 acquired via vaccinated cohorts would allow the gradual transition to the reduced use of enhanced PPE only in some circumstances, when dental patients belong to the low-risk category (Table 2), with expected COVID-19 negative status and mainly in the areas with low, steadily declining COVID-19 rates. At the end of 2020, All Wales Clinical Dental Leads COVID-19 Group prepared a report for the Welsh Government, recommending Standard Operating Procedure (SOP) for dental services as part of de-escalating the COVID-19 response, which includes COVID-19 risk categories of patients [43].

## 5. The Inevitably Altered and Adjusted Dental Facilities

According to the Occupational Safety and Health Administration (OSHA) professional hazard pyramid for COVID-19, DCWs are placed in the ‘remarkably high exposure risk’ category during AGP. Therefore, the attempt to reduce bio-aerosol microdroplets will be likely to remain a top priority for the dental profession even after widespread COVID-19 vaccination [44]. Apart from a standard recommendation, such as routine use of high-volume suction, installing fit-for-purpose air change ventilation systems (AVSs), with negative pressure operating rooms (NPRs) and airborne infection isolation rooms is deemed imperative for dentists to be able to treat patients safely during the pandemic. However, most health authorities across the world have declared this to be an arbitrary requirement if the patient history and physical examination results suggest an asymptomatic patient [44]. Based on the existing data and taking into account the practicability of proposed alterations in dental surgeries, we believe that AVSs and NPRs will become the main element of COVID-19 adjustments to curb the risk of infection and shall allow full re-installation of routine dentistry.

The instant removal of microparticles and microdroplets by incorporating AVSs during AGP is equally paramount and cost-effective; therefore, it should be considered mandatory, or at least a highly recommended option in the future. According to the regulatory bodies, it is recommended that treatment rooms, including dental surgeries, should provide minimum ten air changes per hour as inadequate ventilation in the clinical setting increases the risk of SARS-CoV-2 transmission [45]. The number of air changes per hour per surgery affects the decontamination time following AGP and estimation of this number estimation has to be done for each individual surgery. High-efficiency particulate arrestor (HEPA) filters, which can eliminate 99.97% of 0.3 μm particles could be also used to filter the contaminated air in the treatment zone during the pandemic [45] as an alternative option when the installation of AVSs cannot be carried out. However, there a possibility that HEPA filters become a source of microorganisms if there is microbe proliferation in the air filter, also they are difficult to clean and expensive to replace [45]. Therefore, imposing isolation and negative pressure operating rooms should be considered an optimal scenario following widespread vaccination for COVID-19. Predicting future changes, AVSs and NPRs may become mandatory requirements for any AGPs in clinical settings.

**Figure 4:**
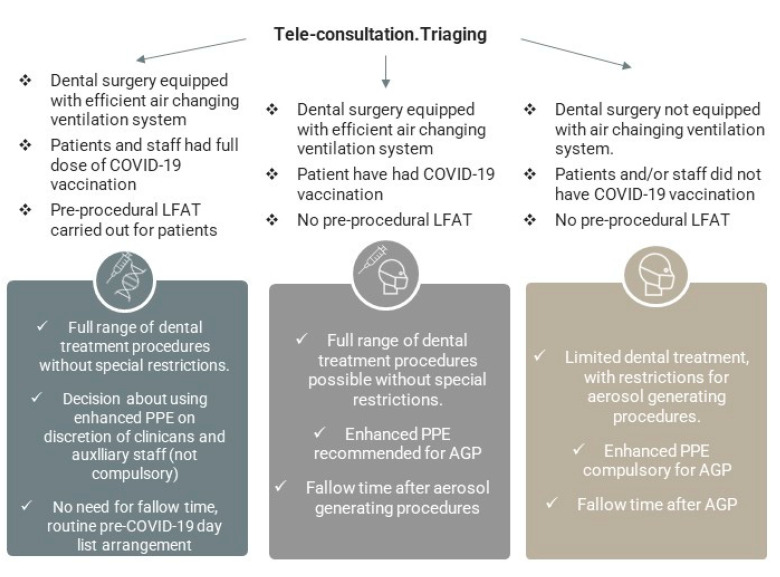
Optimal plan for dental care service resumption subject to adequate SOP, adapted facilities, COVID-19 diagnostics, and immunity status. Regions with fluctuating and unpredictable COVID-19 rate.

The suggested proposal of the decision–action plan for dental providers, based on the presence of in-surgery air changing systems, known COVID-19 vaccination status, and access to LFATs is presented in Figure 4. These different approaches can be practically implemented in a situation when the COVID-19 cases rate fluctuates (new waves), based on the combined AVS’s, COVID-19 rapid testing and vaccine status during the transition phase.

The potential three different pathways with accompanying SOP measures can be executed as part of staged resumption of the dental services. These meaningful scenarios as a response to COVID-19 depend upon economic and social aspects, reflecting specific policies and regulations imposed globally. If broadly utilized, they will alleviate the existing local inequalities in using more advanced anti-COVID-19 measures and shall vastly improve access to routine primary and specialist dental interventions, predominantly in community or hospital services. Considering the advantages of these realistic approaches, dental providers have evidence-based options to support them in restoring activities, although a locally specific fine-tuning would be required.

The knowledge of aerosol-generating mechanisms and way of COVID-19 spread has contributed to the subsequent correction of the mishaps occurring in daily dental practice [46]. It has been acknowledged that there is urgent need for change in the current architecture of dental clinics. A recent study [47] has suggested a modification in clinics’ design, clearly separating the treatment areas (where patients are in direct contact with the dentist) from non-treatment areas (waiting areas, reception, lavatory, rest rooms, and office space). Preferably, dental laboratories and X-ray rooms should be located in the proximity of the treatment areas, while the reception area and consultation/waiting rooms should be near the main entrance [47]. Similarly, short-treatment units, e.g., in dental schools, should be designed next to the consultation rooms, whereas units used to provide lengthy procedures should be located far from the main clinical hub space. In both cases, the treatment areas are required to be large enough and equipped with sufficient ventilation and efficient air change systems to prevent exposed surfaces from contamination during AGPs [47].

In addition to current rigorous infection control measures, a broad implementation of negative pressure dental operating rooms seems to be a priority, regardless of the level of dental care (primary vs. secondary) or geographical location. This can be combined with the modification of the design, and materials used for contact surfaces in dental clinics should also be re-considered. The incorporation of ‘smart’, bioactive materials, metal and/or metal oxide nanoparticles into surfaces could be considered as part of the new measures since they effectively reduce contamination of the environment [46]. In relation to the specifics of dental treatments, decontamination of removable prosthodontic or orthodontic appliance should also be taken into account since they are demonstrated to be a source of intra-oral microbiota transmission [39]. As a result, dental clinics set up will need to undergo essential modifications and adjustments shortly, in order to protect dental teams and patients more effectively.

## 6. The Predicament; Combined SOP/COVID-19 Test/Vaccine Approach

**Figure 5:**
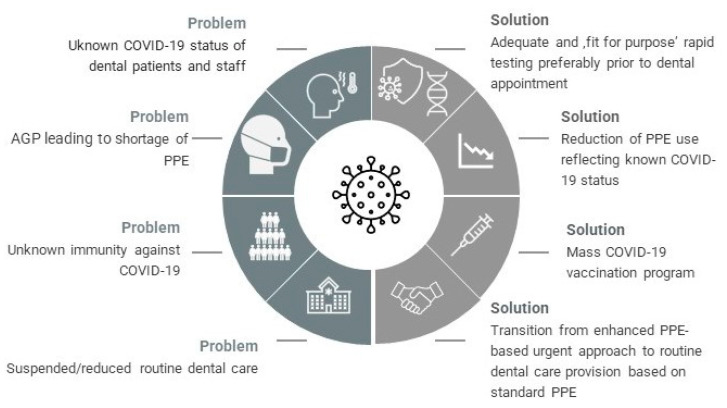
Resuming dental practice during the COVID-19 pandemic. Challenges and solutions.

Substantial reduction to limit the risk of exposure to both DCWs and patients is paramount. While the proposed actions would involve increased costs and levels of complexity for DCWs, they impose a serious task for management [48]. Treatment would become more time-consuming and costly. Subsequently, the number of daily treatments to be performed would be limited [48]. These major disruptions to dental healthcare should be perceived as a red flag, indicating a need to change the way we manage patients. Challenges around the development and implementation of new measures, along with practical solutions, can be efficiently managed with the support of policymakers, dental healthcare commissioners, and agenda for constructive changes (Figure 5). Therefore, the adequate and updated SOP, a gradual relaxation of special measures, high vaccine uptake with effective protection, and rapid testing are essential to restore dental care provision, by minimising future outbreaks.

This is the main task for governments, local authorities, health care commissioners, and professional bodies to support the full recovery of healthcare services logistically or financially [48]. Wide-scale application of these measures will provide a safe environment for treatment, securing the wellbeing of clinicians and patients. Unfortunately, the subdued response to dynamic changes in pandemics, as well as resumption of services reflects the lack of appropriate policies, deficit of expertise, funding, and infrastructure for rapid tasting [48]. We should continue to investigate further the ways to reduce the infection risk by adopting specific cross-infection protocols and by using innovative products.

**Figure 6:**
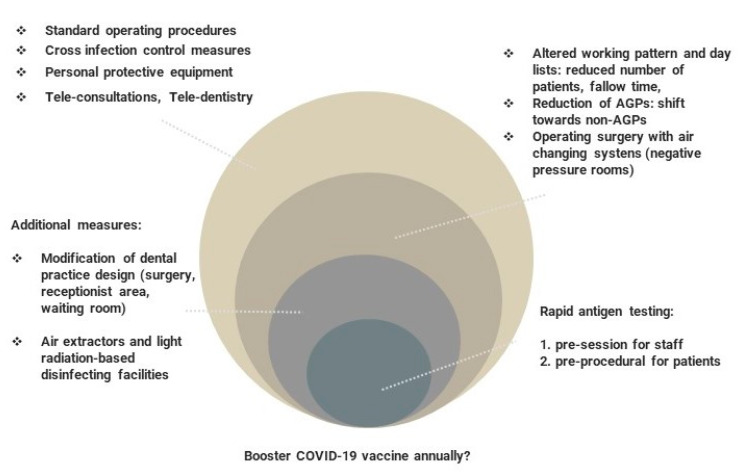
Prioritization of mitigation measures in dental care. The staged reinstallation of dental practice during and after the COVID-19 pandemic.

It has been suggested that the resulting situation can be compared to the universal ‘hierarchy of needs’. The more advanced hierarchical needs can only be considered when the basic needs have been fulfilled [49]. Similarly, in today’s new reality, crucial aspects are pushed to the top of the COVID-19 dental practice and general healthcare ‘hierarchy of needs’. DCWs should all be aware of this set of special measures, based on recent health authority guidelines. Four levels of tasks, from basic to advanced measures are presented in Figure 6. The introduction of each basic task should be performed permanently and effectively, which is necessary for the implementation of the next stage of tasks. Conversely, as we move away from the general policies, more emphasis should be placed on achieving more comprehensive and precise tasks, such as using rapid testing for COVID-19. Nevertheless, this stage would be inefficient without first considering the basic, universal measures.

## 7. Long-Term Prediction: Being Prepared for Health and Dental Care Disruption

**Figure 7:**
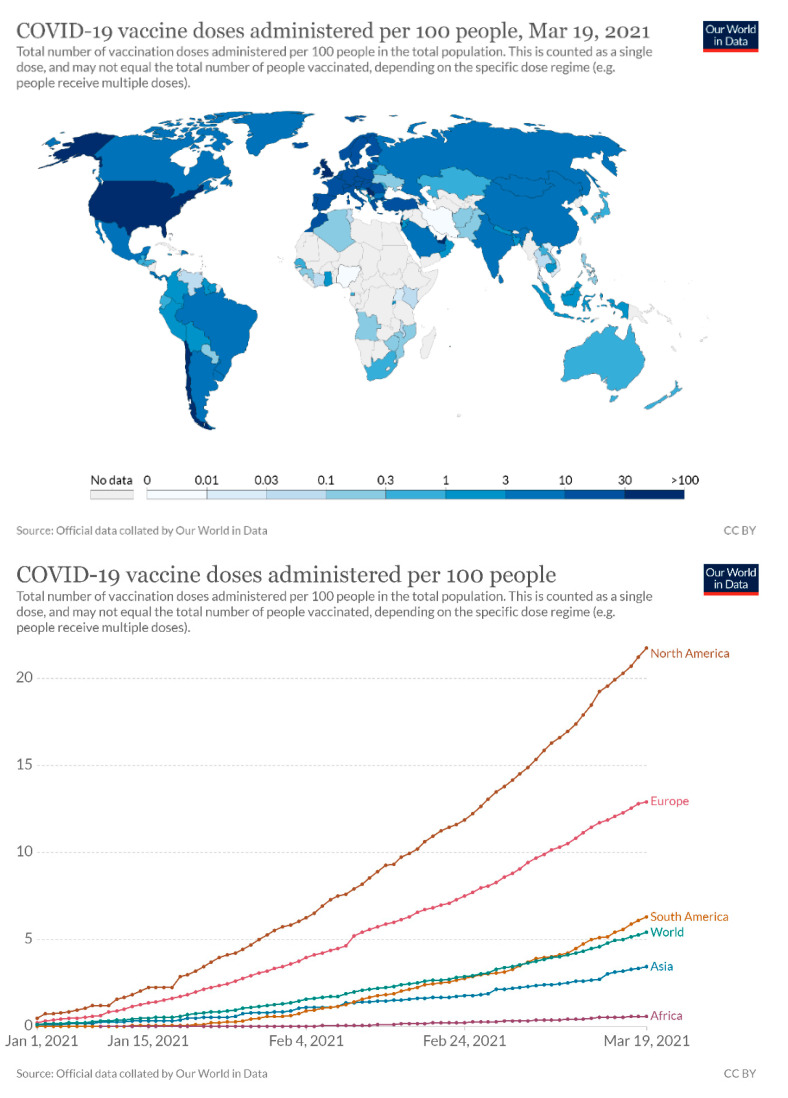
The rates of COVID-19 vaccine administration in different countries and continents up to 19 March 2021 (https://ourworldindata.org/covid-vaccinations).

Expanded worldwide ‘pan-vaccination’ is considered crucial to acquire herd immunity, enabling special measures to be relaxed and safe resumption of dental activity, whilst limiting the risk of serious respiratory complications. As of 10th March 2021, there were more than 200 vaccines in pre-clinical and clinical development [50], including 21 in phase three trials, six in the early/limited use phase, and six approved for full use [51]. Equal distribution of the COVID-19 vaccine worldwide seems currently to be a major, unresolved, and time-consuming challenge [52,53]. However, this tendency related to the disproportionate vaccine delivery to different regions is escalating (Figure 7). Therefore, during the transition phase, standard and essential precautions based on SOP should be always considered to reduce the exposure of both DCWs and patients to SARS-CoV-2.

Until worldwide mass-scale vaccination is completed, preventive and diagnostic measures must be maintained in primary and secondary services based on national guidelines in order to suppress transmission of the virus. Looking ahead, oral inoculation is deemed a complementary and scalable option in preventing COVID-19 as oral vaccine formulation would ease vaccine distribution, manufacturing, and administration, which in turn shall expand immunization, especially in resource-limited populations [54]. We believe that local authorities responsible for healthcare are committed to ensuring equitable access to efficacious, affordable COVID-19 vaccines and diagnostics.

## 8. The Three-Tier System of Dental Services Reinstallation in Regions Where Testing System Is Not Available

Predicting the nearest future, dental practice regarding SOP and AGP will consider primarily the regional COVID-19 cases rate, which should be monitored regularly. This is especially important as rapid testing for COVID-19 may not be available in a vast majority of regions, due to cost and infrastructure implications. Therefore, better support for developing countries is needed, including a transfer of new POCT technology and increase in testing capacity. In addition, a valid consent aspect exists, concerning personal preferences or obstacles. According to the risk assessment protocol, individuals who decline pre-procedural testing fall into the ‘moderate risk category’. In these circumstances, the decision about the practice SOP involving rapid testing should rely on assessed COVID-19 risk for patients (Table 2), along with their vaccination status.

Interestingly, the COVID-19 vaccination rate as such may not be sufficient to influence decisions about reducing the use of enhanced PPE for AGPs. The new SOP designed for dental providers during post-pandemic era will have to address this concern. The proposed three-tier resumption system intends to incorporate real-time data associated with regional COVID-19 rates, the number of COVID-19 positive cases per local population, the percentage of inoculated individuals, as well as the incidence of infections with new variants.

**Figure 8:**
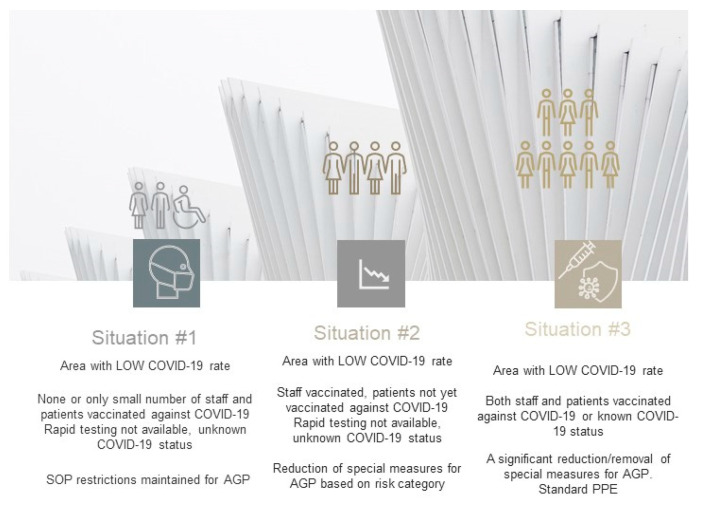
The proposal three-tier system in areas with low COVID-19 cases rate and testing not available. Options for change applicable for SOP depending on local COVID-19 cases rate and population immunity status. Stable, predictable COVID-19 rate locally.

The first, the least favorable situation involves the area with a low COVID-19 rate (R index below 1 generally), where no or only a limited number of staff and patients had been fully vaccinated, and rapid tests are not available. In this case, due to the unknown COVID-19 status of patients, preventative measures concerning AGPs should be still maintained and continued. In the area where the COVID-19 rate is low and predictable, and the staff has been vaccinated, but the dental patients have not been vaccinated yet, and rapid testing is not implemented, it is possible to reduce SOP restrictions for AGP. The decision should rely on the predicted negative COVID-19 status following the patient’s risk assessment (Table 2) and dental procedures can be performed with fewer restrictions applied for AGPs. This approach would lead to a reduction in enhanced PPE usage. Finally, in an ideal scenario, both DCWs and patients are fully vaccinated in the area with low and steady COVID-19 rate. Likewise, this would minimise the risk of COVID-19 transmission in dental teams, with changes to be introduced in the protocols concerning AGP and reduction of special preventative measures (Figure 8).

**Figure 9:**
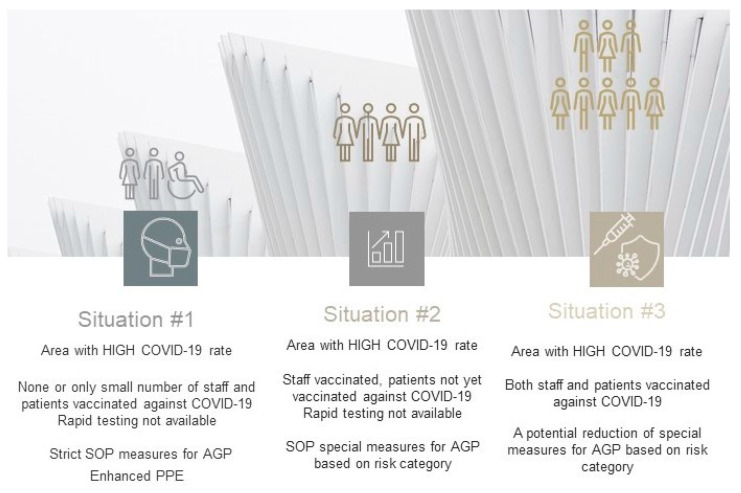
Proposed three-tier system in areas with high COVID-19 cases rate (new waves, local outbreaks) and rapid testing not available. Options for change applicable for SOP in future depending on local COVID-19 cases rate and population immunity status. Unstable COVID-19 rate.

In the regions with high COVID-19 rates, rapidly spreading infections, e.g., local virus resurgence, the proposed management of dental services must consider a stricter approach and risk assessment procedures (Figure 9, Table 2). The high rate of spreading COVID-19 cases spread locally may preclude permanent resumption of medical and dental services. In areas with rapidly escalating rates of COVID-19 where only limited cohorts have been vaccinated, it would be necessary to reinforce the strict SOP and limit dental care provision to urgent and/or emergency cases only.

Undoubtedly, whilst these options have potential limitations due to dynamic development of the pandemic, and heterogenicity between different geographical regions, they should be interpreted with caution as they may disproportionally disadvantage a considerable number of dental care providers. In the future, medical and dental care provision, resumption of healthcare services, and the need for using enhanced PPE could be graded by health policymakers using the ‘traffic lights system’ determined by COVID-19 rate, the proportion of the vaccinated population, and locally calculated risk of SARS-CoV-2 contraction. This approach is especially relevant during a pandemic transition time as DCWs fatigue increases while COVID-19 spread continues. It has to be highlighted, the feasibility of developing and implementing such a ‘multi-pillar’ protocol should be monitored, and pre-validated on a wider scale, adapting to highly specific characteristics of regional dental and medical workforces.

Our proposal for a tier-based system might vastly contribute to Public Health surveillance agendas on national levels as a response to the pandemic, monitoring the COVID-19 prevalence in local communities. This can be achieved by exchanging sets of various of COVID-19 epidemiological data from a wide range of sources. The enhanced PPE vigilance within a specific community will depend upon this information, shared with healthcare providers. Finally, this should be interpreted in the light of the dynamically changing COVID-19 pandemic and acceleration of vaccination programs. Although the proposed protocols and tiers are likely applicable by any healthcare provider, further scrutiny is required.

## 9. Conclusions

To the best of our knowledge, this is the first report of the easy-to-employ proposal for dental services reinstallation based on COVID-19 rates, testing accessibility, and vaccination rate. The equal distribution of the vaccine for COVID-19 around the world and in specific groups, including front-line personnel and vulnerable individuals, remains a global challenge. Before effective vaccination for various cohorts of society and different SARS-CoV-2 variants are broadly implemented, the use of adapted facilities, dentistry-specific delivery systems, altered working patterns, and operating protocols will be essential. It is necessary to pursue and deploy reliable point-of-care diagnostic countermeasures. Regardless of the expected vaccination success, the nearest future of dentistry may rely on the core operating protocols, regional COVID-19 rates, and consequently the predicted risk of infection.

Altogether, the utilization of fit-for-purpose standard operating procedures and engaging reliable rapid testing in routine practice are recommended for screening of any possible infections. Dental practice will likely emerge from the pandemic permanently changed, with new perspectives. Short and long-term re-instalment plans need to be arranged by the local authorities and government bodies.

## Figures and Tables

**Table 1:** The main advantages and disadvantages of rapid lateral flow antigen tests.

Advantages	Disadvantages	Limitations
Simplified use and performance in outpatients’ clinic. Suitable to be used at the workplace, including dental surgery.	Suboptimal relative sensitivity (wide range 74–94%), false-negative results if low viral antigen load in a sample. A negative result does not eliminate the possibility of SARS-CoV-2 infection	Positive results do not rule out co-infections with other pathogens
Short reaction time, rapid results within 5–20 min.	Test’s sensitivity depends on the active phase of viral infection	Specimens collected after five days of illness are more likely to be negative compared to RT-PCR assay
A small amount of sampling material required	Positive results need to be verified by molecular RT-PCR testing	Negative results are not intended to rule out other viral or bacterial infections
High specificity about 100%. High accuracy 98–99%	Results may not correlate with the clinical history and other diagnostic methods performed on the same specimen	The amount of antigen in a sample may decrease as the duration of illness increases
Can detect both viable and non-viable material	Cost	
To be used for the qualitative detection of SARS-CoV-2 antigens from nasal swabs, throat swabs or sputum samples only		
Equally sensitive for different SARS-CoC-2 variants		

**Table 2:** COVID-19 risk categories of patients.

Low Risk	Medium Risk	High Risk
Triaged or clinically assessed patients with no symptoms, with known recent COVID-19 status, or patients who recently have self-isolated, shielded.	Triaged or clinically assessed patients with no symptoms, with no known recent COVID-19 contact	Untriaged persons present for assessment or treatment (status and symptoms unknown)
Persons who have recovered from COVID-19 recently and had few days without any symptoms	Asymptomatic persons with unknown infectious status	Symptomatic or suspected COVID-19 persons
Patients who had a negative COVID-19 PCR test within 72 h of treatment and had been self-isolating from the test date	Asymptomatic persons who declined testing	History of contact with COVID-19 individual, they are waiting for test results

Traffic light’ system, adapted and modified, Standard Operating Procedure for the Dental Management of Non-COVID-19 Patients in Wales, v.1.01, Wales, UK.

## Data Availability

Not applicable.
